# GP50 as a promising early diagnostic antigen for *Taenia multiceps* infection in goats by indirect ELISA

**DOI:** 10.1186/s13071-016-1915-5

**Published:** 2016-12-01

**Authors:** Xing Huang, Jing Xu, Yu Wang, Cheng Guo, Lin Chen, Xiaobin Gu, Weimin Lai, Xuerong Peng, Guangyou Yang

**Affiliations:** 1 Department of Parasitology, College of Veterinary Medicine, Sichuan Agricultural University, Chengdu, 611130 China; 2 Chengdu Agricultural College, Chengdu, 611130 China; 3 College of Science, Sichuan Agricultural University, Ya’an, 625014 China

**Keywords:** *Taenia multiceps*, GP50, Immunofluorescence, Indirect ELISA

## Abstract

**Background:**

Coenurosis is caused by coenurus, the metacestode of *Taenia multiceps*, which mainly parasitizes the brain and spinal cord of cattle, sheep and goats. To date, no widely-approved methods are available to identify early coenurus infection.

**Methods:**

In this study, we identified a full-length cDNA that encodes GP50 (*Tm*GP50) from the transcriptome of *T. multiceps*, and then cloned and expressed in *E. coli.* The native proteins in adult stage and coenurus were located via immunofluorescence assays, while the potential of recombinant *Tm*GP50 protein (r*Tm*GP50) for indirect ELISA-based serodiagnostics was assessed using native goat sera. In addition, we orally infected 20 goats with mature *T. multiceps* eggs. Praziquantel (10%) was given to 10 of the goats 45 days post-infection (p.i.). Blood samples were collected for 17 weeks p.i. from the 20 goats and anti-r*Tm*GP50 antibodies were evaluated using the indirect ELISA established here.

**Results:**

The *Tm*GP50 contains an 897 bp open reading frame, in which signal sequence resides in 1 ~ 48 sites and mature polypeptide consists of 282 amino acid residues. Immunofluorescence staining showed that native *Tm*GP50 was localized to the microthrix and parenchymatous zone of the adult parasite and coenurus, and the coenurus cystic wall. The indirect ELISA based on r*Tm*GP50 exhibited a sensitivity of 95.0% and a specificity of 92.6% when detecting GP50 antibodies in sera of naturally infected goats and sheep. In goats experimentally infected with *T. multiceps*, anti-*Tm*GP50 antibody was detectable from 2 to 17 weeks p.i. in the control group, while the antibody fell below the cut-off value about 3 weeks after praziquantel treatment.

**Conclusion:**

Our results indicate that recombinant *Tm*GP50 is a suitable early diagnostic antigen for coenurus infection in goats.

## Background

Coenurosis is caused by the metacestode of the tapeworm *Taenia multiceps*, known as coenurus. The coenurus mainly parasitizes the brain or spinal cord of ungulates, especially sheep and goats, causing lethal neurological symptoms [1–8]. *Taenia multiceps* induced coenurosis occurs almost all over the world, causing enormous economic losses to the livestock industry and also threatening human health through potentially fatal zoonotic infections [1–8].

Glycosylphosphatidylinositol-anchored proteins (GPI-APs) are attached to the plasma membrane by a glycosylphosphatidylinositol anchor [9–11]. GPI-APs are structurally and functionally diverse and play vital roles in numerous biological processes [9]. Hancock et al. [12] showed that the *T. solium* GP50 protein (*Ts*GP50) is a diagnostic antigen for cysticercosis by Western blot methods. Additionally, a Falcon assay screening test-enzyme-linked immunosorbent assay (FAST-ELISA) [13, 14] and QuickELISA [15, 16] based on *Ts*GP50 have been successfully established to diagnose *T. solium* cysticercosis. These methods have high sensitivity and specificity.

Because the clinical symptoms of coenurosis vary and goats infected with *T. multiceps* do not show obvious clinical symptoms in the early stage of infection [17, 18], it is difficult to diagnose coenurosis. Various clinical manifestations increase the complexity of diagnosis. Thus, it is urgently necessary to develop a diagnostic approach which is both specific and practically acceptable [19]. In many areas, the prevalence of cerebral coenurosis is believed to be underestimated because of the lack of reliable diagnostic methods [20]. In the present study, we tested the tissue distribution of *Tm*GP50 in the parasite, and developed an indirect ELISA assay based on recombinant *Tm*GP50 for the early diagnosis of *T. multiceps* infection in goats.

## Methods

### Animals

Two female 70-day-old New Zealand white rabbits were obtained from a rabbit farm in Sichuan Province, China. Twenty healthy goats were obtained from a goat farm at the Laboratory Animal Center of Sichuan Agricultural University.

### Parasites

Adult *T. multiceps* were collected from artificially infected dogs. Coenuri were isolated from the brains of naturally infected goats. All samples were washed three times with sterile saline solution and then stored in liquid nitrogen until use.

### Cloning, expression and purification of recombinant *Tm*GP50

Total RNA extraction from coenurus and synthesis of cDNA were performed as described previously [21]. Based on transcriptome data from *T. multiceps* and the GP50 sequence of *T. solium* (GenBank accession no: AY214922.1), the gene sequence of *Tm*GP50 was amplified by PCR using primers 5′-CCG GAA TTC GAA AAT GCC CCA A-3′ and 5′-CGG CTC GAG TCA CAA AAC CAT TGG TAT CA-3′ with *Bam*HI and *Xho*I restriction enzyme sites (underlined). The construction of expression vectors and purification of the recombinant protein were also performed as described previously [21].

### Sequence analysis

The open reading frame was predicted using ORF Finder (http://www.ncbi.nlm.nih.gov/gorf/gorf.html), the presence of a signal peptide was assessed using SignalP 4.1 at the Center for Biological Sequence Analysis website (http://www.cbs.dtu.dk/services/SignalP/). The molecular weight and pI values of the predicted protein were calculated using Compute pI/Mw at ExPASy (http://web.expasy.org/protparam/). Phylogenetic analysis was performed using MEGA 5.1 software.

### Sera

Naturally infected positive serum samples were collected from farms in Sichuan Province, China, including 20 serum samples from goats naturally infected with *T. multiceps*, seven serum samples from goats naturally infected with *Cysticercus tenuicollis* and eight serum samples from sheep naturally infected with *Echinococcus granulosus*. Negative sera (36 samples) were collected from cestode-free goats (confirmed by autopsy), from which 24 samples were used to determine the cut-off value and 12 samples were used to test the specificity of the indirect ELISA established in this study. Three-hundred and forty serum samples were collected from 20 goats artificially infected with *T. multiceps* eggs, and the serum were collected once a week for 17 weeks post-infection (p.i.) until the end of the experiment. All sera were stored at –20 °C until use.

### Preparation of polyclonal antibodies against r*Tm*GP50

Rabbit serum was collected before immunization to provide a reagent for negative controls. Two female New Zealand rabbits were immunized three times at 2-week intervals by hypodermic injection of r*Tm*GP50. For the first immunization, 200 μg r*Tm*GP50 emulsified with an equal volume of Freund’s complete adjuvant (Sigma, California, USA) was injected subcutaneously. The second and third injection was given by mixing 100 μg r*Tm*GP50 with an equal volume of Freund’s incomplete adjuvant. Two weeks after the final injection, rabbit antiserum was collected. The antibody titer was determined by indirect ELISA established in the present study. Besides, immunoglobulin G (IgG) was collected and purified by HiTrap ProteinA (Bio-Rad, Hercules, CA, USA) for subsequent immunohistochemistry.

### Immunoblotting and immunofluorescence

For immunoblotting analysis, total protein extracts of coenurus and purified r*Tm*GP50 were separated by SDS-PAGE and subsequently transferred onto polyvinylidene fluoride membranes (Boster, Wuhan, China). The remainder of the immunoblotting procedure was performed as described previously [21]. For immunolocalization studies, procedures were performed as described elsewhere [21], with modified rabbit anti-*Tm*GP50 antibodies (1:400) and fluorescein isothiocyanate (FITC)-conjugated goat anti-rabbit IgG (1:300; Boster).

### Development of indirect ELISA

The optimal antigen dilution and serum dilution for ELISA were determined by using a standard checkerboard titration procedure. Briefly, r*Tm*GP50 protein was two-fold serially diluted to six different concentrations (3.148, 1.574, 0.787, 0.396, 0.197 and 0.098 μg/ml) in 0.1 M carbonate buffer (pH 9.6), and then 100 μl were added to 96-well ELISA plates and incubated at 4 °C overnight. The plates were washed three times with 0.01 M phosphate-buffered saline containing 0.05% Tween 20 (PBST) and blocked with 5% skim milk (100 μl per well) for 90 min at 37 °C. Serial two-fold dilutions of the positive and negative serum samples (100 μl; diluted in PBS), ranging from 1:5 to 1:80, were added to the wells and incubated for 1 h at 37 °C. After additional washing, HRP-labelled rabbit anti-goat IgG was diluted to 1:3000 in 0.01 M PBST and then added to each well (100 μl). After 90-min incubation at 37 °C and washing again, 100 μl tetramethylbenzidine (TMB) were added to the plates and incubated for 15 min in darkness at 37 °C. The absorbance was then determined in a microplate reader at 450 nm after the reaction was stopped. We chose the optimal working conditions which gave the highest P/N value and for which the OD_450_ value for positive serum was around 1.0. We used serum samples from 24 coenurus negative goats to determine the cut-off value in the optimal conditions, which was calculated as the mean OD_450_ plus three standard deviations (SD).

### Sensitivity and specificity of indirect ELISA

The sensitivity and specificity of the indirect ELISA were calculated as follows: sensitivity (%) = ELISA positive × 100/true positive; specificity (%) = ELISA negative × 100/true negative. Serum samples from sheep infected with *E. granulosus* and goats infected with *C. tenuicollis* were used to evaluate the cross-reactivity of r*Tm*GP50.

### Experimental infection of 20 goats with *T. multiceps*

Twenty healthy adult goats were randomly divided into a drug treatment group and a control group (10 goats per group). All goats were orally given an average of 5500 mature, viable *T. multiceps* eggs. Forty-five days p.i., the drug treatment group was treated with 10% (w/v) praziquantel by intramuscular injection (at a dose of 70 mg/kg of body weight, once each day for 2 days). Blood samples from the 20 goats were collected once a week for 17 weeks p.i.

### Surveillance of the anti-*Tm*GP50 antibody in goats artificially infected with *T. multiceps*

The anti-*Tm*GP50 antibody of a total of 340 serum samples, collected from 20 goats artificially infected with *T. multiceps*, was detected using the indirect ELISA established in this study. In addition, as a parallel control, these samples were subjected to the indirect-ELISA based on recombinant *Taenia multiceps* acidic ribosomal protein P2 (r*Tm*P2) to detect the anti-*Tm*P2 antibody at the same time (the results have been published [21]).

### Statistical analyses

ELISA data are presented as the mean value ± standard deviation (SD). Statistical analyses were performed with one-way ANOVA (LSD test) for comparison between groups using SPSS version 20.0 (SPSS Inc., Chicago, IL). *P*-values < 0.05 were considered to be significant.

## Results

### Sequence analysis, expression and reactivity of r*Tm*GP50

**Fig. 1:**
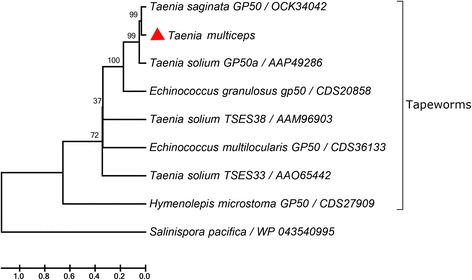
Phylogenetic analysis of *Tm*GP50 protein with homologues of other taeniid species. Node numbers represent bootstrap values

**Fig. 2:**
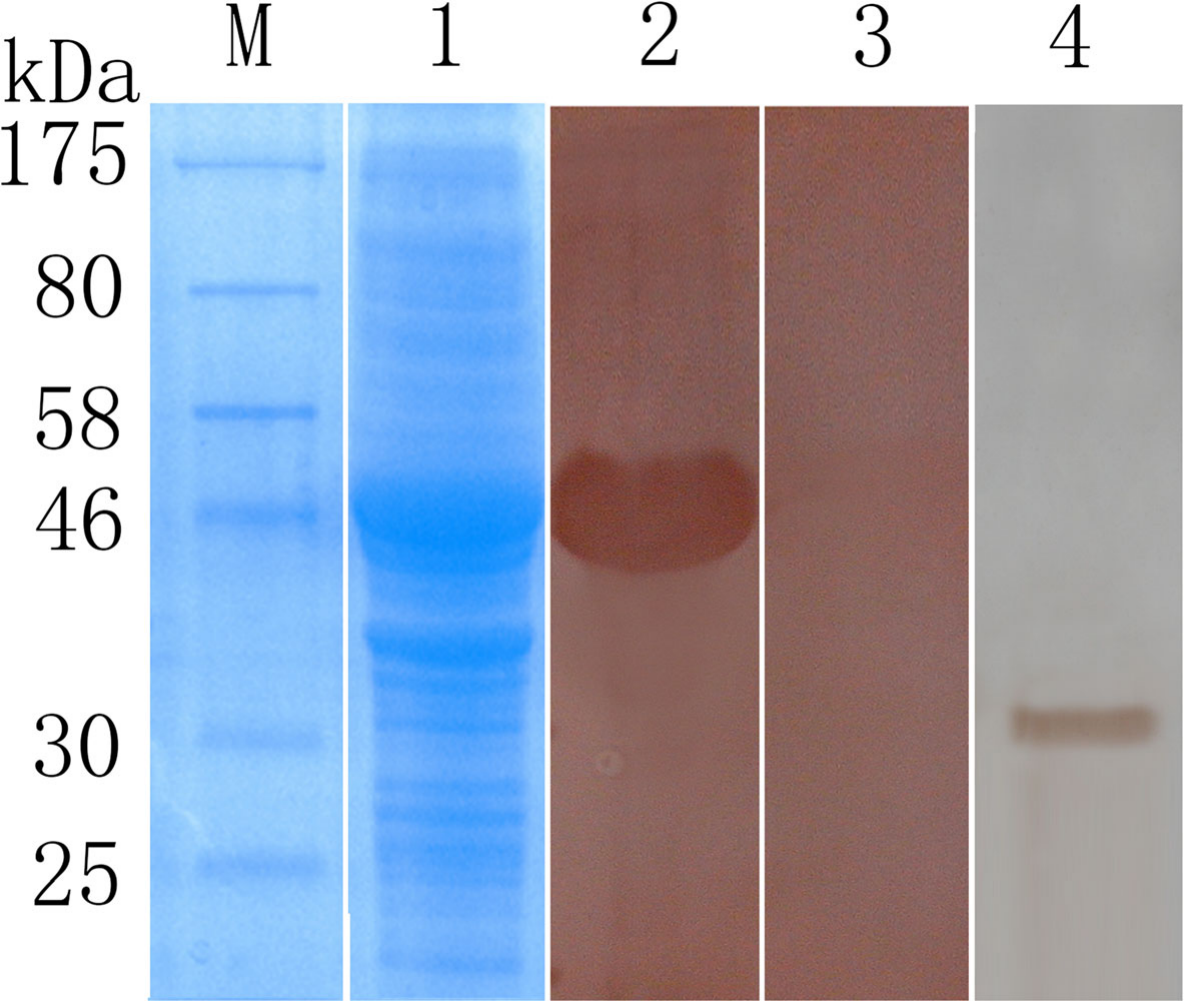
Immune recognition of recombinant *Tm*GP50 and native *Tm*GP50 by Western blotting. Lane 1: SDS-PAGE of extracts of IPTG-induced *E. coli* cells expressing *Tm*GP50; Lanes 2, 3: immune recognition of r*Tm*GP50 using serum from a *T. multiceps* infected goat (Lane 2) and naïve goat serum (Lane 3); Lane 4: Western blot analysis of crude extracts of *T. multiceps* probed with goat immune serum

*Taenia multiceps* GP50 contains an 897-bp open reading frame, encoding a signal sequence from resides 1–48 bp and a mature polypeptide of 282 amino acid residues. The protein (exclude the signal sequence) had a predicted molecular weight of 31.46 kDa, and a pI of 8.23. The amino acid sequence of *Tm*GP50 (contains the signal sequence) shared 94% identity with GP50 from *T. asiatica*, 87% identity with GP50 from *T. solium* and 69% identity with GP50 from *E. granulosus*. A phylogenetic NJ tree based on the amino acid sequences of GP50s showed that all the tapeworm protein clustered together (Fig. 1). Recombinant *Tm*GP50 was expressed in *Escherichia coli* and was present in inclusion bodies at ~51 kDa (Fig. 2, Lane 1). Excluding a ~20 kDa epitope tag fusion peptide, r*Tm*GP50 had a molecular weight of approximately 31 kDa, which was similar to that predicted from its amino acid sequence. Immunoblotting using serum from a goat naturally infected with *T. multiceps* showed a single positive band at ~51 kDa, indicating this recombinant protein had strong reactivity (Fig. 2, Lane 2). Total crude protein extract from *T. multiceps* was blotted with anti-r*Tm*GP50 rabbit serum and a band of approximately 31 kDa was observed (Fig. 2, Lane 4).

### Immunolocalization of native *Tm*GP50 protein in adult *T. multiceps* and coenurus

**Fig. 3:**
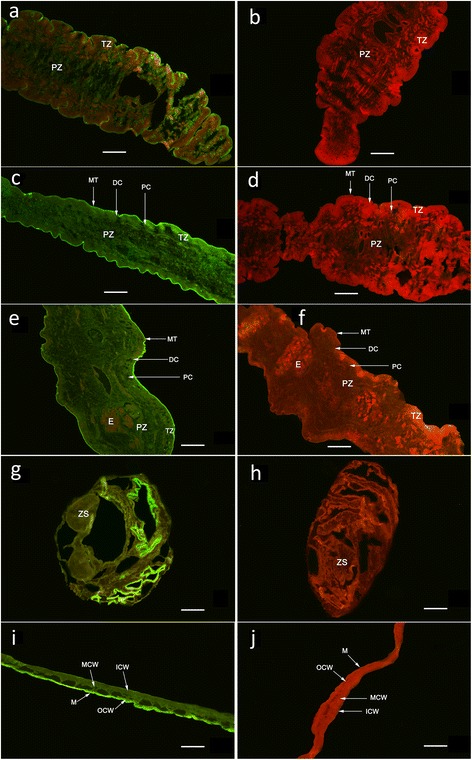
Immunolocalization of *Tm*GP50 in the larval and adult stages of *T. multiceps*. The green fluorescent colour shows the location of *Tm*GP50 protein. *Tm*GP50 protein in the adult tapeworm (**a**-**f**) and coenurus (**g**-**j**) was immunofluorescently-labelled using specific anti-r*Tm*GP50 IgG (**a**, **c**, **e**, **g** and **i**) or negative serum (**b**, **d**, **f**, **h** and **j**), followed by FITC-conjugated goat anti-rabbit IgG. **a** and **b** represent cephalomeres of adult tapeworm; **c** and **d** represent immature segments of adult tapeworm; **e** and **f** represent mature segments of adult tapeworm; **g** and **h** represent cephalomeres of coenurus; **i** and **j** represent the cystic wall of coenurus. The magnification of all the images is × 100. *Abbreviations*: MT, microthrix; DC, distal cytoplasm; PC, perinuclear cytoplasm; TZ, tegument zone; PZ, parenchymatous zone; E, eggs; ZS, zone of scolex; ZN, zone of neck; M, microtriches; OCW, outer layer of cystic wall; MCW, middle layer of cystic wall; ICW, inner layer of cystic wall. *Scale bars*: 100 μm

Fluorescence immunohistochemistry showed that native GP50 protein was highly localized to the microthrix and parenchymatous zone of both the adult parasite and the coenurus; it was also widely distributed in the cystic wall of the coenurus (Fig. 3). No signal was detected in the negative controls.

### Establishment of indirect ELISA

The optimal conditions for indirect ELISA were: antigen concentration 0.197 μg/well and serum dilution 1:10 (Table 1), with an optimal P/N value of 2.76. Twenty-four negative serum samples were tested in these conditions, and the cut-off value was calculated as 0.581 (mean + 3SD): the mean absorbance was 0.329 and the standard deviation was 0.0840 (data not shown). All tests were performed with three replicates. Serum samples with OD_450_ ≥ 0.581 were therefore defined as coenurus antibody positive, otherwise they were considered coenurus antibody negative.

**Table 1:** Determination of the optimal protein coating concentration and serum dilution for indirect ELISA

Antisera at different dilutions	OD_450_ values of antigen at different coating concentrations					
	0.098 μg	0.197 μg	0.394 μg	0.787 μg	1.574 μg	3.148 μg
1:5 (P)	1.043	1.104	1.116	1.160	1.216	1.274
1:5 (N)	0.417	0.442	0.551	0.720	1.035	1.036
1:10 (P)	0.807	**0.921**	0.888	0.877	0.952	0.973
1:10 (N)	0.307	**0.333**	0.440	0.607	0.753	0.771
1:20 (P)	0.584	0.626	0.616	0.741	0.853	0.926
1:20 (N)	0.255	0.264	0.300	0.425	0.594	0.605
1:40 (P)	0.427	0.429	0.506	0.507	0.592	0.645
1:40 (N)	0.200	0.243	0.260	0.332	0.468	0.527
1:80 (P)	0.367	0.393	0.398	0.405	0.392	0.581
1:80 (N)	0.187	0.203	0.215	0.242	0.244	0.337

*Abbreviations*: *N* positive serum, *P* negative serum

*Note*: Figures in bold represent the optimum conditions for this indirect ELISA method

### Sensitivity and specificity analysis of the indirect ELISA

**Fig. 4:**
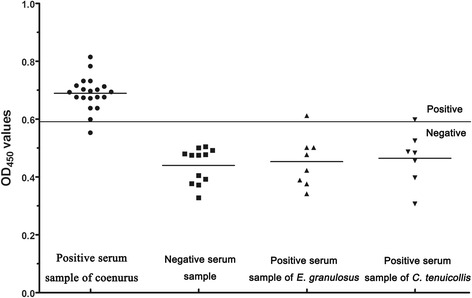
Sensitivity and specificity of the indirect ELISA. The bold horizontal line represents the cut-off value (0.581). Statistically significant differences between *T. multiceps*-positive sera and the other sera samples, including *C. tenuicollis*-positive sera, *E. granulosus*-positive sera and healthy goat sera were tested by one-way ANOVA using SPSS version 20.0 (ANOVA: *F*_(3,43)_ = 147.97, *P* < 0.0001). No difference was noted among the *C. tenuicollis*-positive, *E. granulosus*-positive and healthy goat sera samples

Nineteen out of 20 serum samples from goats naturally infected with coenurus were detected as positive, indicating that the sensitivity of the indirect ELISA was 95.0% (Fig. 4). We observed cross-reactivity with one *C. tenuicollis*-positive goat (*n* = 7), one *E. granulosus*-positive sheep (*n* = 8) and no healthy goats (*n* = 12); thus the specificity of the ELISA was 92.6% (25/27) (Fig. 4). There were statistical differences observed in the ELISA values between the *T. multiceps*-positive sera and the other sera samples, including *C. tenuicollis*-positive goat sera, *E. granulosus*-positive sheep sera and healthy goat sera (ANOVA: *F*_(3,43)_ = 147.97, *P* < 0.0001). No difference was noted among the *C. tenuicollis*-positive, *E. granulosus*-positive and healthy goat sera samples.

### Anti-*Tm*GP50 antibody in goats experimentally infected with *T. multiceps*

**Fig. 5:**
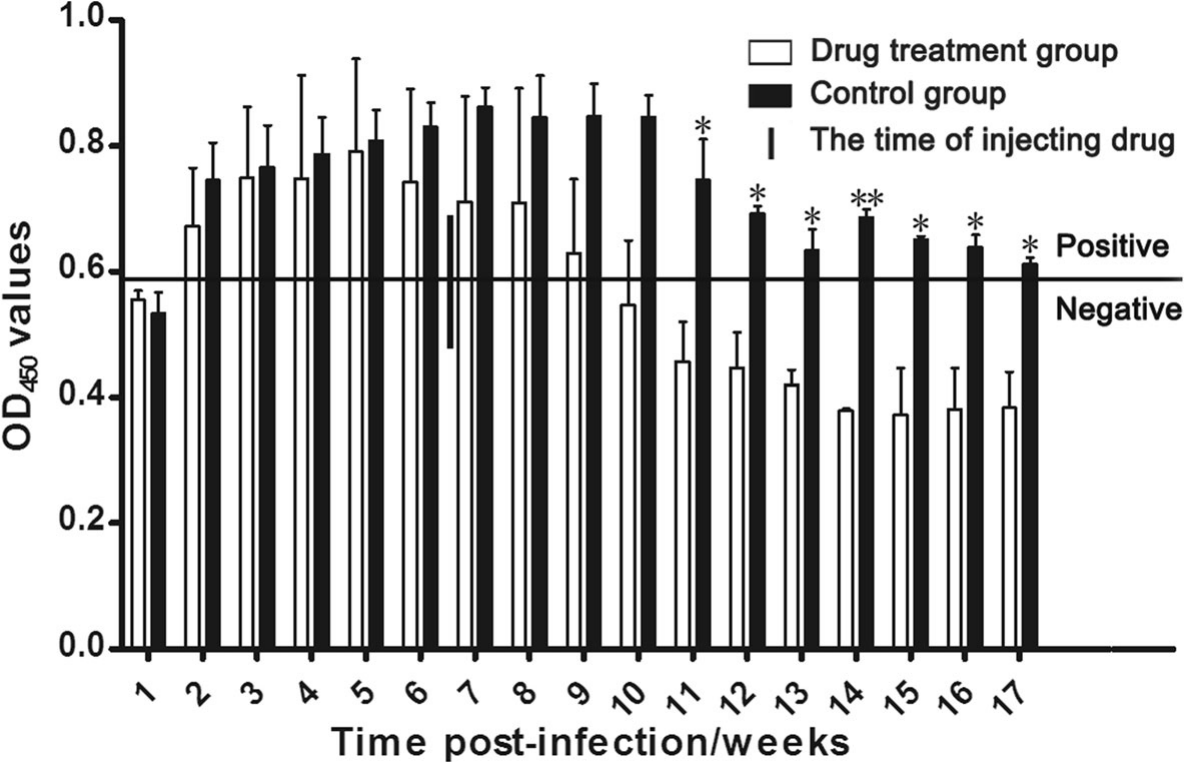
Serum antibody profiles induced by *T. multiceps* infection of goats. The bold horizontal line indicates the cut-off value (0.581). Asterisks indicate statistically significant differences of anti-*Tm*GP50 antibody between the drug treatment group and control groups (*P < 0.05; **P < 0.01), and the error bars represent the standard deviation, SD. Forty-five days post-infection (as indicated in bold *black* vertical line), the drug-treatment group was treated with 10% (w/v) praziquantel by intramuscular injection at a dose of 70 mg/kg of body weight, once each day for 2 days

Figure 5 shows the levels of detected serological antibodies after goats were experimentally infected with *T. multiceps*. From 2 weeks p.i. until the end of the experiment, the control group was positive for serum antibody to *Tm*GP50. Two weeks p.i., the drug-treated group was also positive for serum antibody to *Tm*GP50 (OD value > the cut-off value of 0.581), but by around 10 weeks p.i. (about 3 weeks post-injection of praziquantel), the antibody value detected in the drug-treated group dropped below the cut-off value (OD < 0.581) until the end of the experiment (at 17 weeks p.i.). Furthermore, the anti-*Tm*GP50 antibody level of drug-treated group and control group showed no significant difference for the former 10 weeks p.i., but showed significant difference ever since 11 weeks p.i. (One-way ANOVA, *P* < 0.05).

## Discussion

In recent years, studies concerning parasite GP50 proteins have mainly focussed on *T. solium*, and confirmed that the *Ts*GP50 protein is a valuable diagnostic marker for cysticercosis [12–16, 22]. In this study, we cloned and expressed *Tm*GP50 from coenurus for the first time. Sequences analysis indicated that *Tm*GP50 has high similarity to *Ts*GP50 at the DNA and amino acid levels. Immunofluorescence showed that the *Tm*GP50 protein was highly localized to the microthrix and parenchymatous zone of both the adult parasite and the coenurus, as well as being widely distributed in the cystic wall of the coenurus, which is in agreement with the characteristics of GPI-APs [9–11].

Medical imaging technology such as magnetic resonance imaging (MRI) and computed tomography (CT) has been used for the diagnosis of coenurosis in humans and animals [23–25]. Morphological abnormalities in the skull, and even the volume of cysts, can be tested in sheep and goats infected with coenurosis by MRI [26, 27]. Because of their high positive predictive value, MRI and CT scans are considered to be the best methods to diagnose coenurosis, but it is very difficult to adapt these methodologies for use with farm animals and they are very costly [28]. In addition, a precondition for successful application of MRI and CT technology is that the coenurus has localized in the central nervous system of the host and formed large cysts, so MRI and CT cannot be used to detect coenurosis in the early stage of infection.

Molecular biology approaches have also been used for the diagnosis of *T. multiceps* infections. Oryan et al. [19] demonstrated that the DNA of *T. multiceps* presents in the cerebrospinal fluid (CSF) of sheep and goats with this disease, and this can be diagnosed by amplification of the *cox1* gene. This method suggested that PCR can be used to amplify parasite DNA from the CSF and is valuable for the accurate identification of coenurosis cases. However, the tedious operation required for the collection of the CSF has limited the employment of this PCR method in clinical practice.

Although different traditional serum methods including ELISA [29, 30], Dot-ELISA [31], indirect haemagglutination assay and dot immunogold filtration assay [32] have been developed to diagnose coenurosis, the antigens used in these assays were natural worm extracts and therefore cannot be produced commercially. Compared with natural worm antigen-based ELISA, indirect ELISA based on recombinant proteins has many advantages including antigen source stability and high reproducibility. To date, indirect ELISA assays based on the recombinant antigens *Tm*7 and heat shock protein 70 have been successfully established to diagnose coenurosis [33, 34], but the early diagnostic value of these antigens has not been explored. Huang et al. [21] successfully established an indirect ELISA assay to diagnose coenurosis based on the recombinant antigen *Tm*P2, and anti-*Tm*P2 antibody was detectable three weeks p.i.

Early diagnostic methods based on excellent antigens are important for the control of coenurosis. The excellent serodiagnostic value of the GP50 antigen has been confirmed in *T. solium* cysticercosis [12–16, 22]. Various serodiagnostic methods based on the GP50 antigen have been established for the diagnosis of *T. solium* cysticercosis, including Western blotting [12], FAST-ELISA [13, 14] and QuickELISA [15, 16]; the sensitivity and specificity can reach > 90%. The present study established an indirect-ELISA for goat coenurosis using recombinant *T. multiceps* GP50 protein as the capture antigen and had high sensitivity (95%) and specificity (92.6%) when compared with the results of necropsy. Moreover, the antibodies could be detected two weeks p.i., which is 7 days earlier than using the indirect-ELISA developed based on *Tm*P2 [21]. The anti-*Tm*GP50 antibodies dropped below the cut-off value about three weeks post-injection of praziquantel and did not exceed the critical value thereafter. Therefore, we can also use this indirect-ELISA to evaluate the efficacy of drug treatment of coenurosis.

## Conclusions

In this study, anti-*Tm*GP50 antibodies can be detected from two weeks post-infection, indicating that recombinant *Tm*GP50 is a suitable early diagnostic antigen for coenurus infection in goats, and the indirect ELISA based on r*Tm*GP50 will be a promising method for the early diagnosis of coenurosis in goats.

## Abbreviations

cDNA: Complementary DNA
p.i.: Post-infection
r*Tm*GP50: Recombinant *Taenia multiceps* GP50
*Tm*GP50: *Taenia multiceps* GP50
